# Social Licking in Pregnant Dairy Heifers

**DOI:** 10.3390/ani5040404

**Published:** 2015-11-24

**Authors:** Grazyne Tresoldi, Daniel M. Weary, Luiz Carlos Pinheiro Machado Filho, Marina A. G. von Keyserlingk

**Affiliations:** 1 Animal Welfare Program, Faculty of Land and Food Systems, University of British Columbia, 2357 Main Mall, Vancouver, BC V6T 1Z4, Canada; E-Mails: gtresoldi@ucdavis.edu (G.T.); dan.weary@ubc.ca (D.M.W.); 2 LETA—Lab of Applied Ethology and Animal Welfare, Department of Zootechny and Rural Development, Federal University of Santa Catarina, Rod. Admar Gonzaga, 1346, Itacorubi, Florianópolis SC 88034-001, Brazil; E-Mail: pinheiro.machado@ufsc.br

**Keywords:** allogrooming, affiliative behavior, cattle welfare, grazing, competition.

## Abstract

**Simple Summary:**

Social licking is often associated with good animal welfare, but little is known about this behavior in cattle. Licking behavior was compared in heifers housed indoors *versus* on pasture. Licking frequency was four-fold higher when heifers were indoors. However, when considering all social interactions recorded (licking and aggressions) licking events represented about 10% of all interactions regardless of housing. This behavior happened more frequently between heifers that were observed more repeatedly in close vicinity of each other. Provision of smaller indoor floor spaces likely brought animals into closer proximity thus facilitating social interactions.

**Abstract:**

Housing affects social behaviors, such as competition, but little work has addressed affiliative behaviors. This study compared social licking (SL) in pregnant heifers housed indoors (in a free-stall barn) *versus* outdoors (on pasture), and relationships with competition, feeding and physical proximity to others. Six heifer groups were observed during two six-hour-periods in both treatments. The total number of social events (SL and agonistic interactions) was four times higher when heifers were housed indoors compared to pasture (546 ± 43 *vs.* 128 ± 7 events/group; *P* < 0.05). SL as a ratio of the total number of social events was similar in the two treatments (12% *vs*. 8% of interactions, free-stall and pasture, respectively; *P* > 0.05). Housing did not affect how the SL bout was initiated and terminated, the duration, the body part licked and behavior preceding licking (*P* > 0.05). Animals in close proximity showed higher rates of SL (*P* < 0.0001) but not agonistic interactions (*P* > 0.05). A previous agonistic event did not predict occurrence or the role of heifers in the following licking event. The higher stocking density indoors likely resulted in increased social interactions.

## 1. Introduction

Social licking is defined as the act of one individual licking the body of another [1]. This behavior is routinely observed at birth when the dam licks her offspring [2], at courtship when the male licks a female in estrus [3], but it also occurs in other contexts between animals of the same sex and age [4].

A variety of functions for this behavior have been proposed. For example, recipients of social licking may benefit from improved coat hygiene [1]. Some studies have also speculated that licking plays a role on the formation and maintenance of social bonds [4,5,6], maintenance of group cohesion [7,8] and reducing social tension associated with agonistic interactions [9,10,11,12]. Social licking is thought to occur more frequently between related animals [6,13,14] including animals that are closer in age [6]. Proximity may be associated with a bond between animals [5,13,15,16,17], and individuals in physical proximity for other reasons may be more likely to engage in social licking. Comparing animals in different housing conditions that vary in space provided per animal, and analyzing individual relationships between animals engaged in social licking, may provide further insights on the role of this behavior in young cattle.

The way in which animals are housed is also thought to influence the social interactions, including agonistic interactions [18]. Indoor housing typically provides less space and more opportunities for cattle to compete for resources, such as lying stalls, feed and water. Previous work has shown that reducing space availability or increasing stocking density can increase competition for feed [11,19,20] and lying stalls [21]. To date, with the exception of an unreplicated study [22], no work has assessed the effects of housing on social licking.

The objective of this study was to compare social licking in dairy heifers housed indoors (in a free-stall barn) *versus* on pasture. Free-stall housing is common in North America [23], but pasture access is perceived to provide certain advantages to the cows [24]. The systems differ in many ways including stocking density and how resources, such as feed and water, are accessed, all of which may influence social licking. We also considered the effects of agonistic interactions, physical proximity to others, and other behaviors on the frequency of social licking.

## 2. Experimental Section

The experiment took place during June and July 2011 at The University of British Columbia Dairy Education and Research Centre (Agassiz, BC, Canada). We used 48 Holstein heifers 18 ± 1.8 months (mean ± S.D.) of age and 134 ± 44 days pregnant. All heifers were managed according to the guidelines of the Canadian Council of Animal Care [25] and were approved by UBC’s Animal Care Committee. Animals were acclimatized to the pasture as a single herd for 15 d before the experiment began and then randomly allocated to 6 groups of 8 heifers each.

Each group of heifers was tested in both pasture and free-stall conditions using a crossover design. Groups were tested sequentially in pairs, with one group randomly assigned to one starting treatment and the other group assigned to the alternate treatment. Treatments were applied for 11 d (7 days of habituation followed by 4 days of data collection) and then switched to the alternate treatment for a second 11-d period.

Free-stall pens were similar to those described by Val-Laillet and collaborators [11] designed to house 12 heifers at a time (117 m^2^ or 14.6 m^2^ per animal, in this experiment). Animals accessed the feed bunk via a headlock barrier (width 60 cm, center to center). There was at least one headlock and one deep bedded sand stall per animal. Heifers were fed a total mixed ration (TMR) formulated according to the National Research Council (NRC) recommendations for growing heifers [26]. Fresh feed was provided daily at approximately 08:00 h and feed was pushed towards the pen three times a day (at 11:00, 18:00 and 22:00 h). Water was provided ad libitum from a single water bin.

On pasture, animals were kept in a 5000 m^2^ paddock (625 m^2^ per animal) enclosed using electric fencing. The field was seeded with Festulolium (*Festuca arundinacea* x *Lolium spp*. cross), *Dactylis glomerata* L. and *Trifolium repens* L. Pasture was the only source of feed. Water was provided ad libitum from a single water trough. All heifers were returned to the pasture after the end of the experiment.

### 2.1. Data Collection

Two observers recorded the behavioral data between 6:00 h and 12:00 h on two separate days during each treatment. On the 8th and 10th experimental day the observations occurred on pasture, and on the 9th and 11th day in the free-stall barn, all by visual direct observation [27]. In both treatments, Observer 1 always recorded the social interactions (social licking and agonistic interactions) using the software Observer XT version 10 (Noldus Information Technology Wageningen, The Netherlands), while Observer 2 always recorded the behavior (posture and activity) of every animal, and its two closest neighbors, using an instantaneous scan sampling technique with a 10-min interval [27,28].

Social licking was identified by repetitive back-and-forth tongue movements behavior performed by one heifer (the groomer) in direct contact with another (the recipient) [11]. Nipping behavior (*i.e.*, small biting movements), a common social behavior in horses [29], was also observed and noted as social licking. For each licking event the groomer and the heifer licked (*i.e.*, recipient) were identified. In addition, specific behaviors, such as the body part licked, whether or not the event was solicited or ended following a forced termination, and bout length, were recorded for all events as described on Table 1.

All agonistic interactions, including displacement and non-physical interactions (threats), and the identity of the instigator (the animal gaining access to the resource or starting the agonistic interaction) and the victim (the one who moved away, who did not react or who avoided the other animal involved) were recorded as described by Hurnik and collaborators [30]. The social status of each animal was estimated using a sociometric matrix described by Kondo and Hurnik [31]. The matrix was calculated based on the total number of wins and losses for each individual in relation to every other animal in the group. The sum of interactions within each pair provided a dominance value for each individual [32]. The difference between the highest and the lowest dominance index in each group was divided into three and used to define dominant (or high-ranking), intermediate, and subordinate (or low-ranking) individuals [32].

Animals within one body length or less were considered to be in close proximity. The number of observations that each heifer was observed in close proximity with every other in the group was calculated as a ratio of the total number of times that heifer as observed in close proximity to any other heifer in the group.

The posture and activity of each animal was recorded as: lying (recumbent) or standing up, feeding (animal with the mouth below (not visible) or at the level of the grass or TMR), ruminating (chewing with lateral jaw movements with the head in line with the body or raised up so that the head was above the mid line of the body), drinking (animal with the lips immersed in the water and neck movements indicative of swallowing), idle (not engaged in any apparent behavior), and other (any behavior other than those described above).

**Table 1:** Definitions of social licking measures recorded using live observations of heifers (*n* = 6 groups) housed indoors in a free-stall barn or outdoors on pasture.

Variables	Description
Groomer	The animal that licks the body of another animal
Recipient	The animal that has its body licked
Body part licked	Head, neck, front part of the body (i.e. all body parts from thoracic vertebrae to chest) or back part of the body (from lumbar, sacral and coccygeal vertebrae to abdomen) as described by Val-Laillet *et al*. [11]
Solicited social licking	Animal has head lowered, chin stretched under the head of another animal, sometimes accompanied by head butts ^a^
Forced termination	Groomer stops licking the recipient after receiving a physical interaction from the recipient or another animal (butt or push)
Duration	Length (in time) of a licking event that was initiated when the tongue touches the body surface of the other animal and ended when the tongue stops touching it. When breaks were <30 s events were compiled into the same bout as suggested by Val-Laillet *et al*. [11]

^a^ Adapted from Sato *et al*. and Laister *et al*. [9,33].

### 2.2. Data Analysis

Mixed models (Proc Mixed is SAS, version 9.2, SAS Institute, 2009) were used to test the effect of treatment (pasture *vs*. free stall) on the total number of social interactions, social licking (as a ratio of total number of interactions), the number of animals involved in social licking events, and the duration of bouts. In all cases the group was used as the experimental unit (*n* = 6). Friedman’s test was used to compare how social licking events were initiated and terminated, preferred body part licked and the behavior recorded before social licking began to the nearest 10-min scan observation. Descriptive analysis was used to analyze the relationship between social licking and any preceding agonistic event. We used all possible pair combinations of heifers in both housing conditions (n = 336 or 56 potential pairs of groomers-recipients/group) to correlate social licking frequency (both executed and received) with social rank, number observations spent in proximity, and the number of agonistic interactions executed and received.

## 3. Results and Discussion

The total number of social events (social licking and agonistic interactions) was four times greater when heifers were housed in the free-stall barn compared to pasture: 546 ± 30 *vs*. 128 ± 30 events/group (mean ± SE, *P* < 0.01; Table 2), respectively. However, the number of social licking events as a ratio of the total number of social interactions was not different between housing conditions (*P* ≥ 0.14, Table 2). Treatment also did not affect the duration of licking bouts, how they were solicited, body part licked, or specific behavior recorded before the licking event (Table 2), but did affect how this behavior was terminated. Forced termination ended 30% and 13% of events for pasture and free-stall treatments, respectively (*P* < 0.01, Table 2). Regardless of housing, with the exception of one heifer, all animals were involved in social licking events as groomers, recipients or as both (Table 2).

**Table 2:** Frequency of social licking for groups of dairy heifers (*n* = 6) kept on pasture and in a free-stall barn. Data are presented as means ± SE.

Variables	Pasture	Free-stall	*P*
Total number of social licking events per group	10 ± 10	67 ± 10	0.01
Social licking (% of all social interactions)	8 ± 2	12 ± 2	0.14
Cows acting as (% of animals):			
Groomer	54 ± 7	81 ± 7	0.03
Recipient	56 ± 9	87 ± 9	0.01
Both	60 ± 14	70 ± 14	0.58
Duration (s)	39 ± 14	37 ± 13	0.91
Solicited social licking (% of licking events)	36	29	0.14
Body part licked (% of licking events)			0.09
Head	46	47	
Neck	25	35	
Front	3	7	
Back	25	10	
Behavior pre-licking (% of licking events)			0.19
Grazing or feeding	71	60	
Idle	21	33	
Other	8	7	
Forced termination (%)	30	13	<0.01

To our knowledge, this study is the first to compare rates of social behavior of cattle housed on pasture and indoors. We recognize that there was less space available per animal in the free-stall barn compared to pasture, so differences between the housing treatments may have been due to space availability or any other difference between the systems. However, pasture and indoor housing typically differ in these ways, so we feel that the results are of practical interest. The differences in space available per animal likely explain the greater total number of both agonistic and social licking events observed indoors compared to pasture. Interestingly, no difference was observed when social licking was expressed as a ratio of the total number of social interactions. Other studies have shown that the number of agonistic interactions increases when space per animal is reduced [11,19,20,21,34], but our work is the first to show that social licking events increases when animals are provided less space. In addition, no difference was observed when social licking was expressed as a ratio of the total number of social interactions. This may suggest the ratio of social interactions is similar across housing conditions where resources are not limited, and animals can freely interact to each other.

Regardless of housing condition, the majority of social licking was performed spontaneously as has been described by other authors [9,33]. Although the initiation of social licking bouts is well documented [9,33], our study is the first to clearly describe the entire duration of the event, including termination. The majority of social licking events ended spontaneously, whereas 15% of all events were terminated immediately after the groomer or recipient engaged in displacement behavior, or were alternatively displaced by another animal. We suggest that when a displacement event was initiated by one of the animals involved in the social licking bout, it was done as a way to end the social licking bout. In contrast, we speculate that when a third animal initiates displacement behavior that results in the termination of a social licking event currently underway, this animal is signaling its desire to engage in a social licking event.

In the free-stall barn, the majority of the social licking was directed towards the head followed by the neck; a pattern previously described by others [9,11,33]. On pasture, licking bouts were still preferentially directed towards the head but there was no clear preference for a second preferred body part (neck and back received the same amount of licking). The focus on the head and neck is not surprising given that individuals cannot groom themselves on these regions, emphasizing the possible hygienic function of social licking [35,36]. Previous work has also reported a higher frequency of licking bouts directed at the back part of a cow’s body when the event started without solicitation [33] or when the recipient was lying down [9], but we did not observe these differences in the current study. Independent of housing condition, the most frequent behavioral state recorded before social licking was feeding, suggesting that licking occurs primarily when animals are foraging. This association has been noted in previous work on free-stall housed cattle [11] and on pasture [8,37].

**Figure 1:**
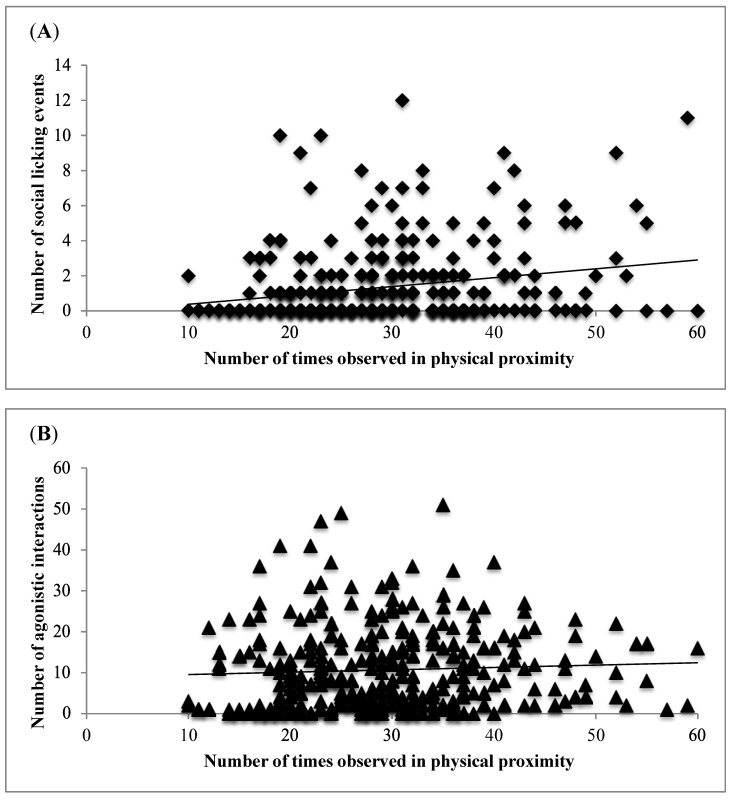
Total number of observations spent in proximity across pairs of pregnant heifers housed in groups (*n* = 6) with 8 heifers per group in relation to (**A**) the total number of social licking events performed by groomers (r = 0.22) and (**B**) the total number of agonistic interactions initiated (r = 0.05).

Across pairs of heifers, regardless of housing, we observed no relationship between social licking, either as the groomer or recipient, and social rank (r = −0.06 and r = 0.01, respectively). We also found no evidence that pairs of heifers that engaged in more social licking as groomers or recipients were more likely to instigate (r = 0.04 and r = 0.09, respectively) or to receive (r = 0.04 and r = 0.05, respectively) agonistic interactions from that partner. Lastly, pairs that spent more time in close proximity to one another were more likely to lick each other than engage in agonistic interactions (Figure 1A,B). Approximately 20% of all social licking events were preceded by an agonistic interaction involving the same pair of heifers. The interval between these interactions averaged 1:43 min (range = 0:03–17:24 min). The role of the heifer in the licking event was independent of the heifer’s role in the previous agonistic interaction regardless of pair combination (same or other). Groomers were the instigators in 47% (thus, victims in 53%) of the agonistic events that took place immediately before the social licking event.

Social licking occurred most often between animals observed more frequently in close proximity. Many authors have described the importance of physical proximity and time spent together in the maintenance of social bonds [3,5,16,17]. Social licking may function to strengthen these relationships [5]. Our results seem to support this hypothesis given that we observed a positive effect on the frequency that pairs spent in close proximity and social licking but not agonistic interactions.

Although social licking has been thought to reduce social conflicts [9,12,38], our results do not support this interpretation. We did not find any effect of social status or agonistic interactions on rates and characteristics of social licking, contrary to other studies [6,11]. In addition, there was no relationship between the pairs of heifers involved in social licking and the previous agonistic interactions. Heifers that were victims of agonistic interactions did not engaged in more licking events as groomers, contrary to the prediction that social licking stabilizes dominance-subordinance relationships [6].

## 4. Conclusions

Our work provides the first evidence that housing conditions affect social licking frequency, however, the ratio of social licking to the total number of social interactions was unaffected by treatment. In addition, this study suggests that physical proximity was positively correlated to social licking events but not with agonistic interactions. This result may indicate that social licking plays an important function in the formation and maintenance of social bonds. We encourage future work to determine if specific heifers are motivated to access a particular partner.
